# Chronic exposure to Interleukin-6 amplifies the response to Toll-like receptor ligands: implication on the pathogenesis of macrophage activation syndrome

**DOI:** 10.1186/1546-0096-9-S1-P210

**Published:** 2011-09-14

**Authors:** Raffaele Strippoli, Francesco Carvello, Roberta Scianaro, Loredana De Pasquale, Marina Vivarelli, Stefania Petrini, Luisa Bracci-Laudiero, Fabrizio De Benedetti

**Affiliations:** 1 Ospedale Pediatrico Bambino Gesù, Piazza Sant’Onofrio 00165 Rome, Italy

## Objective

To analyse whether prolonged exposure to IL-6 affects the inflammatory response induced by TLR ligands.

## Methods

IL-6 transgenic (IL-6TG) or wild type mice were stimulated with different TLR ligands; survival, blood cells counts and biochemical parameters were analyzed. Murine splenic mononuclear cells and peritoneal macrophages from these mice were stimulated with LPS, LTA, poly I:C or CpG. Human macrophages were treated for 4 days in presence of IL-6 and then stimulated with LPS. Inflammatory cytokine expression was measured by ELISA or by RT-PCR. Activation of STAT3, ERK1/2 MAPK, p65 NF-κB was evaluated by Western blot or by confocal analysis.

## Results

Treatment of IL-6TG mice with TLR ligands led to increased lethality with increased levels of IL-1β, TNFα, IL-6 and IL-18. Macrophages from IL6TG mice released higher amount of inflammatory cytokines that was associated with increased phosphorylation of STAT3, ERK1/2 and with increased NF-κB nuclear translocation. Human macrophages treated with IL-6 and then stimulated with LPS showed an increased expression of cytokines and similarly increased signalling pathways activation. IL6TG mice following LPS administration showed increased ferritin and soluble CD25, as well as decrease in platelet and neutrophil counts and in hemoglobin levels compared to WT mice.

## Conclusions

Prolonged exposure to IL-6 in vivo and in vitro leads to an exaggerated inflammatory response to TLR ligands. Hematological and biochemical abnormalities in IL-6 transgenic mice treated with LPS show striking similarities with macrophage activation syndrome.

